# Prevention of Ovarian Hyperstimulation Syndrome: A Review

**DOI:** 10.1155/2015/514159

**Published:** 2015-05-14

**Authors:** Vinayak Smith, Tiki Osianlis, Beverley Vollenhoven

**Affiliations:** ^1^Alice Springs Hospital, Department of Obstetrics and Gynaecology, Alice Springs, NT 0870, Australia; ^2^Monash IVF, 252 Clayton Road, Clayton, VIC 3168, Australia; ^3^Monash Health, Women's and Children's Program, Monash Medical Centre, Clayton Road, Clayton, VIC 3168, Australia; ^4^Department of Obstetrics and Gynaecology, Monash University, Clayton, VIC 3168, Australia

## Abstract

The following review aims to examine the available evidence to guide best practice in preventing ovarian hyperstimulation syndrome (OHSS). As it stands, there is no single method to completely prevent OHSS. There seems to be a benefit, however, in categorizing women based on their risk of OHSS and individualizing treatments to curtail their chances of developing the syndrome. At present, both Anti-Müllerian Hormone and the antral follicle count seem to be promising in this regard. Both available and upcoming therapies are also reviewed to give a broad perspective to clinicians with regard to management options. At present, we recommend the use of a “step-up” regimen for ovulation induction, adjunct metformin utilization, utilizing a GnRH agonist as an ovulation trigger, and cabergoline usage. A summary of recommendations is also made available for ease of clinical application. In addition, areas for potential research are also identified where relevant.

## 1. Introduction

Ovarian hyperstimulation syndrome (OHSS) is encountered in practice as an iatrogenic complication of controlled ovarian stimulation (COS). COS is aimed at producing multiple ovarian follicles during assisted conception cycles in hope of increasing the number of oocytes available for collection. OHSS, however, is characterised by an exaggerated response to this process [1, 2].

The incidence of moderate to severe OHSS is between 3.1 and 8% of in vitro fertilization (IVF) cycles but can be as high as 20% in high risk women [3, 4]. Typically, OHSS is a phenomenon which is associated with gonadotrophin use during COS. There are instances, however, where OHSS has been documented to arise spontaneously either in conjunction with clomiphene or with gonadotrophin releasing hormone use [2, 5]. This review aims to examine the pathophysiology of OHSS and the evidence behind the various methods employed by clinicians to prevent its occurrence.

## 2. Methods

A literature search was carried out on the following electronic databases (until December 2014): MEDLINE, EMBASE, and The Cochrane Central Register of Controlled Trials. Only articles in English were taken into consideration and abstracts were excluded. A combination of text words or Medical Subject Headings (MeSH) terms were subsequently utilized to generate a list of citations: (“OHSS” OR “ovarian hyperstimulation syndrome”) AND (“prevention”). Articles and their references were then examined in order to identify other potential studies which could provide perspective for the following review.

Systematic reviews, meta-analyses, and randomized controlled trials (RCTs) were then preferentially selected over other forms of data where feasible in order to formulate the following review and recommendations.

## 3. Results and Discussion

### 3.1. Pathophysiology

OHSS is theorized to manifest systemically as a result of vasoactive mediators being released from hyperstimulated ovaries. As a result, capillary permeability is increased which causes the extravasation of fluid from the intravascular compartment into the third space. The haemoconcentration which ensues results in complications such as hypercoagulability and reduced end organ perfusion [6, 7].

There is currently no consensus on the exact cause of OHSS. Human Chorionic Gonadotrophin (hCG) exposure, however, is thought to be a critical mediator of the syndrome. This is based on the findings that OHSS does not develop when hCG is withheld as an ovulatory trigger during COS and also that increased hCG exposure is associated with an increased risk of OHSS [8, 9].

The role of hCG can be further elucidated via the two distinct clinical presentations observed in OHSS: the “early” and “late” forms. “Early” OHSS occurs within 9 days of hCG being administered as an ovulatory trigger and reflects the effect of exogenous hCG on ovaries that have already been hyperstimulated by gonadotrophins. “Late” OHSS, on the other hand, occurs more than 10 days after the use of hCG as an ovulatory trigger (in the absence of luteal hCG support) and demonstrates the ovarian response to endogenous hCG produced by the trophoblast [9].

hCG is thought to play a key role in the pathophysiological mechanism of OHSS by mediating the release of vascular endothelial growth factor-A (VEGF-A). VEGF-A, through its interactions with the VEGF receptor-2 (VEGFR-2), promotes angiogenesis and vascular hyperpermeability. Its overexpression, therefore, characterises the increased vascular permeability observed in OHSS [10, 11]. VEGF-A concentrations have been demonstrated to be elevated after hCG administration and in women with or at risk of OHSS [12, 13].

**Figure 1:**
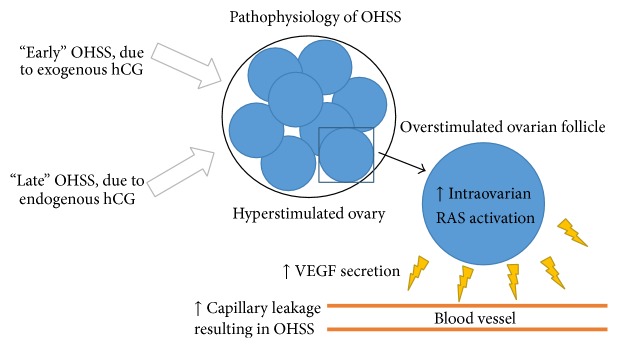
Graphical representation of the pathophysiology of ovarian hyperstimulation syndrome (OHSS).

Another pathophysiological mechanism implicated in OHSS is the intraovarian renin angiotensin system (RAS). The ovarian RAS is involved in regulating vascular permeability, angiogenesis, endothelial proliferation, and prostaglandin release. hCG causes a strong activation of the RAS, evidenced by high renin activity in the follicular fluid of women with OHSS [11, 14]. Overstimulation of this cascade, together with increasing VEGF levels, is postulated to synergistically potentiate OHSS (Figure 1) [15, 16].

### 3.2. Prevention of OHSS

As the old adage goes, prevention is better than cure. As it stands, there is no perfect strategy which completely eliminates OHSS. There are factors however which we can take into consideration in order to reduce its incidence.

#### 3.2.1. Identifying the “At Risk” Woman

Being aware of the risk factors for OHSS will allow clinicians to preempt its occurrence and thereby reduce its incidence during ovulation induction with gonadotrophins.

*(A) Primary Risk Factors*. Preexisting risk factors for OHSS include young age, low body weight, polycystic ovarian syndrome (PCOS), and a previous history of OHSS [3, 17, 18].

Hormonal markers are also increasingly being utilized in predicting ovarian response to stimulation. Anti-Müllerian Hormone (AMH) in particular is a marker which shows much promise. Gnoth et al., in their prospective study of 316 women, have demonstrated that AMH [AMH ≤ 0.18 pmol/L (1.26 ng/mL)] can identify normal responders (≥4 oocytes retrieved) to COS with a success rate of 98% [19]. This predictive capacity extends to identifying women at risk of developing OHSS as well. Using receiver operating characteristics (ROC) curves, Lee et al. have identified a high pretreatment basal AMH concentration [AMH > 0.47 pmol/L (3.36 ng/mL)] as a useful predictor of developing OHSS (sensitivity 90.5%, specificity 81.3%). Moreover, AMH performed better than weight, age, or ovarian response markers in identifying these women [20]. Given its low inter- and intracycle variability, AMH has the potential to become an excellent predictive tool should issues surrounding its validity be completely resolved [21].

Absolute serum oestradiol (E_2_) concentrations, however, have performed poorly in identifying women at risk of developing OHSS. This can mostly be attested to the marked heterogeneity in studies with regard to the threshold E_2_ levels used to define high risk women [8, 22].

Ultrasonographic markers, such as the antral follicle count (AFC), are also another facet worthy of mention in the prediction of OHSS. Available evidence suggests that the AFC is equally predictive of excessive response to COS and OHSS as the basal serum AMH [23–25]. Jayaprakasan et al., in their prospective study of 1012 subjects, noted an AFC ≥ 24 to be correlated with an increased risk of moderate to severe OHSS in comparison to an AFC < 24 (8.6% versus 2.2%) [26]. These findings are mirrored by Delvigne and Rozenberg and Papanikolaou et al. who cite an increased risk of OHSS with an AFC (2–8 mm) ≥ 12. There are, however, variances amongst the studies regarding the definition of what constitutes antral follicles on ultrasound which limits their applicability [3, 27].

*(B) Secondary Risk Factors*. Secondary risk factors examine ovarian response parameters related to COS in the hope of predicting OHSS. During COS, ultrasound and serum E_2_ monitoring are considered to be vital components of surveillance for OHSS. Based on this, parameters such as a rapidly rising E_2_ level, a large number of developing follicles on the day of hCG administration (>14 follicles with a diameter of 11 mm), and a large number of oocytes retrieved have been proposed as risk factors for developing OHSS [17, 28]. None of the above predictors, however, have been shown to be independently predictive of OHSS and can be considered to be moderate at best given the wide variation in cut-off levels being utilized [1, 3, 29].

In combination, however, Papanikolaou et al. in their prospective cohort of 2524 GnRH antagonist cycles have identified the combination of ≥18 follicles on ultrasound (diameter ≥ 11 mm) and E_2_ ≥ 5000 ng/L on the day of hCG trigger to be more useful (sensitivity 83%, specificity 84%) than E_2_ concentrations alone in the prediction of severe OHSS [28].

It also should be noted, however, that women without any risk factors can develop OHSS as there is some degree of hyperstimulation in all stimulation protocols. The possibility of OHSS therefore should always remain at the back of the clinicians mind in any woman undergoing COS [29].

#### 3.2.2. Risk Stratification

Prevention strategies for OHSS are broadly classified as both primary and secondary in nature. Primary prevention classifies a person based on their risk factors into high, normal, or low risk for OHSS, then individualizing treatment regimens to them on that basis. Secondary prevention, on the other hand, focuses on methods used in patients who have displayed an excessive response to ovarian stimulation during a cycle and aims to prevent progression to OHSS [1].

#### 3.2.3. Primary Prevention

In women who are identified as being at a high risk of OHSS, treatment regimens need to be modified in view of curtailing an overexcessive ovarian response.

*(A) Targeting Unifollicular Ovulation*. As previously highlighted, women with PCOS are at an increased risk for OHSS. Since 4–8% of women worldwide have the syndrome, this is a major subpopulation towards whom primary prevention should be directed. The goal of therapy therefore in this subgroup of women is to induce unifollicular ovulation through ovulation induction (OI) and thereby prevent progression to OHSS [30]. With this in mind, aspects which deserve consideration are as follows.

*(i) Reducing the Gonadotrophin Dose*. The best evidence suggests that the minimum gonadotrophin dose should be used for OI given its lower risk of OHSS. This favours a “step-up” regimen over a “step-down” regimen. In the “step-up” regimen, ovarian stimulation is initiated with a low dose of FSH (i.e., 75 IU), which is subsequently increased every 7 days (i.e., 37.5 IU) until an ovarian response is noted (follicle > 10 mm). This dose is then continued until the criteria for an ovulatory trigger are met [2, 18]. This regimen is associated with a lower risk of OHSS, cycle cancellation, and a higher rate of unifollicular development in contrast to other low dose/step-down protocols. In a “step-down” regimen, a higher starting FSH dose is used which is downtitrated based on ovarian response [31, 32].

*(ii) Avoiding Adjunct GnRH Agonist (GnRHa) Utilization*. During OI in women with PCOS, GnRHa is concomitantly administered with gonadotrophins to downregulate the endogenous pituitary secretion of LH in hope of preventing premature luteinisation. This process, however, seems to increase the dose of exogenous gonadotrophins required [1]. In their Cochrane Review, Nugent et al. highlighted the sequelae of this through the higher overstimulation rate (OR 3.15; 95% CI 1.48–6.70). This coupled with the increased cost and additional inconvenience without an increase in pregnancy rates prompted them to make a recommendation against its use [33].

*(iii) Reducing the Gonadotrophin Duration*. There is consensus on the fact that reducing the duration of gonadotrophin exposure reduces the risk of OHSS. One way this is achieved is through “mild” stimulation protocols which delay the administration of FSH till the mid or late follicular phase [1, 34]. Previously, a major issue associated with this was early cycle cancellation due to premature luteinisation and lower pregnancy rates. However, the addition of GnRH antagonists for late cycle suppression of gonadotrophin release has resulted in improved clinical outcomes, a lower risk of OHSS, and multiple pregnancies and made it cost effective as well. On a side note, the pooled data of 3 RCTs have shown mild stimulation to be less effective than conventional “long” regimens in terms of the pregnancy rates per cycle (15% versus 29%) [35–38].

*(iv) Utilising Adjuvant Metformin Therapy*. Metformin is theorized to exert its influence in preventing OHSS by inhibiting the secretion of vasoactive molecules, such as VEGF, during OI and thereby modulates vascular permeability [39]. In the recent Cochrane Review by Tso et al., based upon 8 RCTs with 798 women, it was noted that there was a lower risk of OHSS with metformin use (OR 0.29; 95% CI 0.18–0.49). It was also of note that metformin reduced the risk of OHSS by 63% and increased the clinical pregnancy rate (OR 1.52; 95% CI 1.07–2.15) [40] without an effect on live birth rates. These findings were consistent with an earlier systemic review by Palombo et al., which described a significantly lower OHSS rate with metformin administration too (0.27; 95% CI 0.16–0.46).

Based on the studies, a daily dose between 1000 and 2000 mg at least 2 months prior to COS is recommended for the purpose of preventing OHSS [41–43].

*(v) Utilising Aromatase Inhibitors (AIs) for Ovulation Induction*. AIs, such as letrozole, function by downregulating oestrogen production through inhibition of cytochrome P450 enzymes. This causes an increase in pituitary secretion of FSH which promotes folliculogenesis. In addition, the central negative feedback mechanisms still remain intact, which leads to the theory that it may reduce the incidence of OHSS during OI [44]. A recent Cochrane Review by Franik et al., however, failed to show any difference in OHSS rates through utilization of AIs in contrast to other methods of OI [45].

As such, AIs are not routinely recommended.

*(B) Individualizing IVF Treatment Regimens*. There is increasing evidence to suggest that individualised COS (iCOS) can reduce OHSS and associated cycle cancellations [46, 47]. iCOS entails identifying women at risk of an overexcessive response through various biomarkers, of which the combination of both AFC and AMH seems to be the most promising [24, 48]. The means of COS (e.g., starting FSH dose or tailored GnRH antagonist protocol) can then be decided based on an algorithm of these biomarkers. One example of this can be seen through the study by La Marca et al., where an algorithm was formulated based on age, AFC, and FSH to calculate the FSH starting dose. This algorithm was able to accurately predict ovarian sensitivity and account for 30% of the variability of ovaries to FSH. In addition, it was also a model that had easy applicability in clinical practice [49]. The CONSORT study also serves as another good illustration of this concept, with adequate oocyte yield and good pregnancy rates (34.2%) [50]. Findings from the ongoing multicentre OPTIMIST study will also be welcome in order to shed light on the cost effectiveness associated with iCOS as well [51].

As it stands, however, iCOS shows a lot of promise in curtailing OHSS through tailored COS regimens and seems to be the initial steps towards an ART of the near future.

*(C) Avoiding hCG for Luteal Phase Support (LPS)*. During COS, endogenous LH concentrations are markedly lower due to the negative feedback caused by the supraphysiological progesterone (P_4_) concentrations maintained by the multiple corpora lutea. This results in a shortened luteal phase and poor endometrial receptivity resulting in reduced implantation and pregnancy rates. As such luteal phase support is imperative to improve these parameters [52–54]. hCG, which is similar to LH in its physiological actions, has been used effectively in this scenario. A Cochrane Review, however, noted that it potentiated the risk of OHSS (OR 3.62; 95% CI 1.85–7.06) and also showed no effect on live birth rate (LBR) and clinical pregnancy rate (CPR). In contrast, the use of progesterone (P) halves the OHSS risk while significantly improving the LBR (OR 2.95; 95% CI 1.02–8.56) and CPR (OR 1.83; 95% CI 1.29–2.61) [55].

On the basis of these findings, the routine use of progesterone over hCG is recommended for LPS.

*(D) Considering Alternatives for Triggering Ovulation*. The agent of choice for triggering ovulation should be picked based on the risk of the woman for developing OHSS. No agent, however, completely eliminates the risk of OHSS.

- *Exogenous hCG* has long been used to mimic the ovulatory LH surge. Its long half-life (2.32 days) however causes prolonged luteotrophic effects, multiple corpora lutea development, and higher luteal phase P_4_ and E_2_ concentrations. Hence, given its higher risk of potentiating OHSS it should be either used at the lowest possible dose (i.e., 5000 IU) or altogether avoided in high risk women [29, 56]. It is of note that the use of lower hCG doses as an ovulation trigger, in contrast to the conventional dose of 10,000 IU, has not impacted clinical outcomes but questions do remain over its capacity to reduce the risk of OHSS [57, 58].
- *GnRH agonists (GnRHa)* produce a more tempered and shorter midcycle gonadotrophin surge (24–36 hours) in contrast to hCG by stimulating pituitary LH secretion. Theoretically, this LH surge should just be sufficient to induce ovulation without being prolonged enough to induce hyperstimulation. The available data supports this notion by demonstrating that OHSS is virtually eliminated with GnRHa utilization (in a “freeze all” approach) which mandates its consideration in the high risk woman [59–61]. This however should be taken in the context of the IVF regimen utilized as well. For instance, with the recent increase in proponents of dual trigger regimens (addition of 1–2000 IU of hCG to a GnRHa trigger) for its improved pregnancy and implantation and live birth rates, the propensity for OHSS remains very possible. It should also be noted that OHSS can occur de novo as part of GnRHa triggered cycles but the incidence of this is limited to a handful of case studies [62–64].
- *Recombinant LH (rhLH)* use is also another possible prevention strategy in the high risk woman by attempting to mimic the endogenous LH surge. With a half-life of 10 hours, and a shorter and/or lower LH peak, it is expected that there should be minimal risk of causing OHSS. A Cochrane Review by Youssef et al. however did not show any difference in the risk for severe OHSS between rhLH and urinary hCG. Furthermore, it has also been associated with a lower pregnancy rate and a poor cost benefit ratio. Its routine use therefore cannot be recommended [65, 66].

#### 3.2.4. Secondary Prevention

Secondary prevention is extended to women who have undergone COS and subsequently mounted an exaggerated response. The aim of interventions in these circumstances is to prevent progression to OHSS.

*(A) Coasting*. Coasting is a preventative strategy by which gonadotrophins are withdrawn when a certain E_2_ concentration and/or a critical number of follicles are reached. hCG trigger is subsequently delayed until E_2_ levels significantly decrease or plateau. Once the E_2_ reaches a “safe” level, hCG is administered followed by oocyte retrieval and embryo transfer or freezing depending on the E_2_ concentration. It is generally employed for a period less than 3 days [29, 67].

Coasting is a commonly used first line secondary prevention strategy by clinicians [68]. Question marks remain however about the evidence behind the procedure. D'Angelo et al., in their Cochrane Review, identified 4 RCTs which highlighted that there was no difference in the incidence of moderate and severe OHSS (OR 0.53, 95% CI 0.44−1.08) with coasting. In addition, a lower number of oocytes were retrieved from the coasting group which prompted them to recommend that there was no benefit of coasting in comparison to other interventions [69]. An earlier meta-analysis also came to the conclusion that coasting may decrease the risk of OHSS in high risk women but does not completely prevent it. Coasting, however, seems to have no effect on live birth rates and clinical pregnancy rates [67, 70].

As it stands, there is not much strong evidence to back its routine use and no specific criteria about commencing and discontinuing coasting given the wide heterogeneity in study protocols, control groups, and definition of OHSS classes as well [1, 67].

*(B) Cryopreservation of Embryos*. During cryopreservation, COS and subsequent oocyte retrieval is performed followed by the cryopreservation of embryos. These are then transferred in a subsequent unstimulated IVF cycle where the woman's ovarian response to hCG has normalized [71]. A Cochrane Review only identified 2 RCTs for analysis and came to the conclusion that there was insufficient evidence to support routine cryopreservation [72]. Recent evidence however strongly supports the use of a GnRHa trigger followed by cryopreservation as being the most effective method in preventing OHSS, best illustrated by Devroey and colleagues through their OHSS-Free Clinic [73].

Another dogma which previously surrounded cryopreserved embryos was the lower pregnancy rates in contrast to fresh embryo transfers related to older slow freezing methods [74]. With the advent of modern techniques such as vitrification, however, there is convincing evidence to suggest that cryopreservation has better pregnancy rates (32% increase) than fresh embryo transfer as well [75–77].

Based on these findings, we recommend the use of a GnRHa trigger followed by cryopreservation for averting OHSS.

*(C) Cycle Cancellation*. Cycle cancellation and withholding of hCG are the only definite methods of preventing OHSS [78, 79]. However, it must be taken in context with the high financial impact and psychological distress that it causes to women. It is therefore, in many cases, a last resort for clinicians [1, 29].

#### 3.2.5. Alternative Methods of Prevention

*(A) Colloid Infusion*. Colloid infusions are administered around the time of oocyte retrieval as they are theorized to prevent OHSS by binding to and deactivating the vasoactive mediators of OHSS.

*(i) Albumin*. A Cochrane Review by Youssef et al. noted that there was borderline statistically lower incidence of severe OHSS with albumin utilization but there was marked heterogeneity in the studies (8 RCTs; OR 0.67; 95% CI 0.04–0.40; *I* ^2^ = 62%). A subsequent sensitivity analysis performed after excluding 2 unpublished studies, however, showed no significant alteration in the results (OR 0.75; 95% CI 0.47–1.21) [80]. Another systematic review by Jee et al. also found that intravenous (IV) albumin did not reduce the rate of severe OHSS (RR 0.80; 95% CI 0.57–1.12) and also raised concerns regarding significantly reduced pregnancy rates (RR 0.85, 95% CI 0.74–0.98) [81]. The lack of prevention against severe OHSS was further reiterated in the systemic review by Venetis et al. (OR 0.80; 95% CI 0.52–1.22) as well. In addition, factors such as the possibility of transmission of viral infections (i.e., hepatitis B/C/HIV) and prion disease through albumin as well as its propensity to cause anaphylactic reactions are risks that should not be overlooked [82].

On the basis of these factors, the routine use of IV albumin to prevent OHSS cannot be recommended.

*(ii) Hydroxyethyl Starch (HES)*. HES is a plasma expander that has been mooted as an alternative to albumin as it is nonbiological and therefore negates the above-mentioned risks associated with albumin use. The evidence behind its benefit is certainly more robust as well. The Cochrane Review by Youssef et al. noted that there was a statistically significant decrease in severe OHSS (OR 0.12; 95% CI 0.04–0.40) with HES use without any effect on pregnancy rates (OR 1.20; 95% CI 0.49–2.95) [80].

It must be borne in mind that these findings were based on only 3 RCTs and more compelling evidence should be sought prior to recommending its routine use.

*(iii) Cabergoline*. Cabergoline is a dopamine antagonist which prevents the excessive increase in VEGF mediated vascular permeability encountered with OHSS through its antiangiogenic properties [83]. Tang et al. in their Cochrane Review of 230 women in 2 RCTs found cabergoline to be effective in significantly reducing the incidence of moderate OHSS (OR 0.38; 95% CI 0.19–0.78) with no significant effect on clinical pregnancy rate and miscarriage rates. This protective effect, however, did not extend to severe OHSS, possibly due to the number of studies available for comparison [84]. A recent systemic review by Leitao at el. on the issue, which took 7 RCTs into consideration, has further established its efficacy in preventing the occurrence of moderate and severe OHSS (RR 0.38; 95% CI 0.29–0.51) as well as without a negative impact on clinical pregnancy or oocytes retrieved [85].

Therefore, the use of cabergoline is recommended and it is suggested that treatment be commenced on the day of hCG trigger at a dose of 0.5 mg for 8 days [86].

*(C) Vasopressin Induced VEGF Secretion Blockade*. Amongst the novel therapies being investigated for the prevention of OHSS, the vasopressin V1a receptor antagonist, relcovaptan, has been studied for its ability to inhibit VEGF by modulating vasoconstriction and vascular smooth muscle proliferation. Relcovaptan, in the hyperstimulated rat model, has shown lower concentrations of VEGF-A in the peritoneal fluid and lesser ovarian weight gain significant decreases in the number of corpora lutea in contrast to control groups. Further research in this area remains rather promising and may broaden the management protocols which clinicians have for OHSS in the near future [87].

## 4. Conclusion

OHSS is a complication associated with COS which clinicians have no complete way of preventing at present. Through the various prevention strategies reviewed in this paper (summarized in Table 1), there are avenues by which its incidence can be greatly reduced. This begins with the identification of the “high risk” woman through to the woman who is “at risk” and subsequently initiating the appropriate therapies. It is also an avenue towards which further research initiatives should be directed in a bid to strengthen the preexisting evidence base for available therapies and to develop novel techniques to aid in the prevention of OHSS.

## Figures and Tables

**Table 1:** Summary of recommendations for strategies to prevent OHSS.

Intervention	Recommendation	Effect of intervention	Level of evidence
Reducing gonadotrophin dose	Recommended	“Step-up regimen” has a lower risk of OHSS, cycle cancellation from hyperstimulation, and higher rate of monofollicular ovulation in contrast to other protocols	1b, 4

Reducing gonadotrophin duration	Utilized as clinically appropriate	“Mild” stimulation protocol with GnRH antagonist for late suppression has a lower risk of OHSS and multiple pregnancies and is cost effective	1b
		It also is less effective in terms of pregnancy rates than “long” protocols	1a

Individualized COS (iCOS)	Further research required	iCOS can reduce OHSS rates and associated cycle cancellations. It also produces a significant oocyte yield and good pregnancy rates	1b, 2a

GnRHa as an ovulation trigger	Recommended	GnRHa use virtually eliminates OHSS rates	1b

hCG as an ovulation trigger	Further research required	Lowest dose of hCG does not seem to reduce OHSS rates	2a, 2b, 4

Adjuvant metformin therapy	Recommended	Metformin is associated with a lower risk of OHSS and increased clinical pregnancy rate	1a, 4

Cabergoline	Recommended	Cabergoline reduces the incidence of OHSS without an effect on pregnancy rates	1a

Hydroxyethyl starch	Utilized as clinically appropriate	HES causes a decrease in OHSS without an effect on pregnancy rates	1a

Coasting	Further research required	Coasting does not completely prevent OHSS, is associated with a lower oocyte yield, and has no benefit in contrast to other interventions. The protocols are also very diverse	1a, 4

Cryopreservation	Utilized as clinically appropriate	Cryopreservation alone does not reduce rates of OHSS	1a
		GnRHa followed by cryopreservation virtually eliminates OHSS	1b

Cycle cancellation	Utilized as clinically appropriate	Cancellation completely eliminates risk of OHSS but has a high financial and emotional burden	4

Adjunct GnRHa use	Not recommended	GnRHa use increases the associated costs and rate of OHSS while lowering the pregnancy rates	1a

Aromatase inhibitors for OI	Not recommended	AIs have shown no reduction in rates of OHSS in contrast to other methods of OI	1a

rhLH	Not recommended	rhLH use does not reduce the risk of OHSS and has higher costs and lower pregnancy rates	1a, 1b

hCG for luteal phase support	Not recommended	Progesterone significantly reduces the risk of OHSS with improved clinical pregnancy rates and live birth rates in comparison to hCG for LPS	1a

Albumin infusion	Not recommended	Albumin does not reduce OHSS rates and may cause lower pregnancy rates. There are also associated risks with anaphylaxis and disease transmission	1a

Vasopressin V1a receptor antagonist	Further research required	It appears to reduce the ovarian weight gain and multiple corpus luteum development in OHSS	2b

Glossary for levels of evidence, 1a: systematic review and/or meta-analysis; 1b: ≥one RCT; 2a: ≥1 well-designed controlled study without randomization; 2b: ≥1 well-designed quasi experimental study; 3: ≥1 well-designed descriptive study; 4: committee or expert opinions.
